# Induction of apoptosis and modulation of homologous recombination DNA repair pathway in prostate cancer cells by the combination of AZD2461 and valproic acid

**DOI:** 10.17179/excli2019-1098

**Published:** 2019-07-08

**Authors:** Saman Sargazi, Ramin Saravani, Javad Zavar Reza, Hossein Zarei Jaliani, Shekoufeh Mirinejad, Zohreh Rezaei, Sadegh Zarei

**Affiliations:** 1 Cellular and Molecular Research Center, Zahedan University of Medical Sciences, Zahedan, Iran; 2 Department of Clinical Biochemistry, School of Medicine, Shahid Sadoughi University of Medical Sciences, Yazd, Iran; 3 Department of Clinical Biochemistry, School of Medicine, Zahedan University of Medical Sciences, Zahedan, Iran; 4 Protein Engineering Laboratory, Department of Medical Genetics, School of Medicine, Shahid Sadoughi University of Medical Sciences, Yazd, Iran; 5 Department of Biology, University of Sistan and Baluchestan, Zahedan, Iran

**Keywords:** AZD2461, drug combination, prostate cancer, PTEN, valproic acid

## Abstract

Cancer therapies using defects in homologous recombination (HR) DNA repair pathway of tumor cells are not yet approved to be applicable in patients with malignancies other than BRCA1/2-mutated tumors. This study was designed to determine the efficacy of combination therapy of a histone deacetylase inhibitor, valproic acid (VPA) and a novel PARP inhibitor AZD2461 in both PC-3 (PTEN-mutated) and DU145 (PTEN-unmutated) prostate cancer cell lines. The Trypan blue dye exclusion assay and the tetrazolium-based colorimetric (MTT) assay were performed to measure the cytotoxicity while combination effects were assessed based on Chou-Talalay's principles. Flow-cytometric assay determined the type of cell death. The real-time PCR analysis was used to evaluate the alterations in mRNA levels of HR-related genes while their protein levels were measured using the ELISA method. γ-H2AX levels were determined as a marker of DNA damage. We observed a synergistic relationship between VPA and AZD2461 in all affected fractions of PC-3 cells (CI<0.9), but not in DU145 cells (CI>1.1). Annexin-V staining analysis revealed a significant induction of apoptosis when PC-3 cells were treated with VPA+AZD2461 (*p<0.05*). Both mRNA and protein levels of *Rad51* and *Mre11* were significantly decreased in PC-3 cells co-treated with VPA+AZD2461 while enhanced H2AX phosphorylation was found in PC-3 cells after 12 and 24 hours of co-treatment (*p<0.05*). Our findings established a preclinical rationale for selective targeting of HR repair pathways by a combination of VPA and AZD2461 as a mechanism for reducing the HR pathway sufficiency in *PTEN*-mutated prostate cancer cells.

## Introduction

Prostate cancer (Pca) is found to be a severe health concern and a preceding cause of cancer-related morbidity in men, being accounted for more than 27000 annual death only in the United States (Ferlay et al., 2015[10]; Li et al., 2005[21]). Over the years, several factors such as genetic alterations, epigenetic changes are proved to have impacts on the transformation and progression of Pca (Al Olama et al., 2014[1]). Mutations in phosphatase and tensin homolog (*PTEN*) which regulates PI3‐kinase activity, have been reported in various human malignancies, including melanoma, glioblastoma, endometrial and prostate tumors (Whang et al., 1998[39]). *PTEN*, as a principal tumor suppressor located on 10q23.3, has been discovered to possess various cellular functions, including regulation of HR-mediated genes, particularly *Rad51* (Shen et al., 2007[36]). This phenomenon can be exploited regarding the development of cytotoxic agents in targeted cancer therapies since various cancer cells frequently harbor gene mutations in DNA repair pathways, causing genomic instability (Ashworth, 2008[3]). Besides, DNA repair pathways are well-established molecular defense systems against cancer-inducing factors, and this enables cancer cells to sustain DNA damages caused by chemotherapeutic agents. Different repair systems protect the human genome including homologous recombination (HR) repair, non-homologous end joining (NHEJ) repair, base excision repair (BER), and micro-homology mediated end joining (MMEJ) repair systems (Jackson and Bartek, 2009[14]). As a result of DNA damage, cancer cells activate a complex DNA repair pathway and ultimately if not efficiently repaired, apoptosis (programmed cell death) takes place (Khanna and Jackson, 2001[18]). Accordingly, poly(ADP-ribose)polymerase (PARP) is the primary enzyme responsible for post-translational modification of early DNA damage response-related proteins since they are capable of sensing DNA breaks as P53 and ATM (Herceg and Wang, 2001[13]). So far, 17 members of PARP superfamily have been identified which have critical functions in numerous cellular processes including DNA repair pathways (Liu et al., 2009[22]), telomere shortening, differentiation and cell death (De Vos et al., 2012[8]). Accordingly, cancer cells harboring deletion mutations in Rad51-mediated pathway, including *PTEN*, undergo a hampered DNA damage response (DDR), making them susceptible to DNA disruptions (Bassi et al., 2013[4]). Of the discovered selective inhibitors of DNA repair, PARP inhibitors provided promising outcomes in various cancer types (Javle and Curtin, 2011[15]). The recent investigation revealed that cells deficient in breast cancer susceptibility proteins (*BRCA*) are 100- to 1000-fold more sensitive to PARP inhibition compared to cells expressing *BRCA* (Zaremba and Curtin, 2007[42]). Olaparib, as an oral PARP1/2 inhibitor, was the first FDA approved PARPi which is currently being used for treating individuals with ovarian cancers harboring germline *BRCA1*/*BRCA2* mutations (Kim et al., 2015[19]), but metaplastic breast and ovarian cancer cells displayed intrinsic resistance to this drug (Vaidyanathan et al., 2016[37]). AZD2461, as a next-generation PARP1/2 selective inhibitor, maintained the same pharmacodynamics and pharmacokinetic characteristics of olaparib (Pommier et al., 2016[32]) but differs in the matter of inducing drug resistance by not being a suitable substrate for efflux transporters (i.e., p-gp) (O'Connor et al., 2016[30]). On the other hand, Histone deacetylase inhibitors (HDAC) are promising anti-proliferative/pro-apoptotic agents that have been used for the treatment of many solid tumors. This include suberoylanilide hydroxamic acid (SAHA), trichostatin A, depsipeptide, and valproic acid (VPA) (Marrocco et al., 2007[24]). It is reported that HDAC inhibitors are capable of downregulating DNA repair induced genes of the HR pathway in cells within certain tumors (Konstantinopoulos et al., 2014[20]), a logical rationale for the use of HDAC inhibitors in cancer therapy. VPA has been described to function as an efficacious growth inhibitory agent against prostate tumor cells, capable of inducing a specific response in time- and concentration-dependent manner (Angelucci et al., 2006[2]). 

Monoallelic loss of *PTEN* is presented in 60% of localized Pca cases, while complete loss of *PTEN* is found to be linked to Pca progression (Phin et al., 2013[31]). Both PC-3 and DU145 Pca cell lines are highly resistant to the anticancer agents. Besides being highly invasive, PC-3 cells have a heterozygous germline mutation in *PTEN* and do not express this tumor suppressor (Vlietstra et al., 1998[38]), while on the contrary DU145 cells express high levels of* PTEN* (Porkka and Visakorpi, 2001[33]). Selective drugs interfering with DNA repair pathways in such cancer cells could prove advantageous in combination with conventional chemotherapeutic agents to make improvements in tumor suppression (Lu et al., 2018[23]). Although germline mutations in *BRCA* family genes are contributed to a high risk of breast and ovarian cancers but are infrequent in Pca cells (0.44% for *BRCA1*; 1-2% for *BRCA2*) whereas *PTEN* inactivation is observed in approximately 70% of primary Pca patients (Cairns et al., 1997[5]). 

To this date, no experiment has evaluated the efficacy of combining a HDACi and AZD2461 in cells with such heterozygous genetic background. Herein, it was hypothesized that combination of VPA and AZD2461 would reduce the proliferation of PC-3 and DU145 cell lines by inducing apoptotic cell death by regulating HR repair induced factors (*BRCA1*, *Rad51* and *Mre11*) in both mRNA and protein level. Our findings can provide a rationale for combining these two agents as a promising approach for Pca therapy. 

## Material and Methods

### Chemicals, assay kits, cells and culture conditions

All reagents and biochemicals used in the present *in vitro* study were analytical grades. VPA, AZD2461, and MTT tetrazolium dye was obtained from Sigma-Aldrich, USA. Fetal bovine serum (FBS) was procured from Gibco (Rockville, MD/USA). Annexin-V-FITC Apoptosis kit was purchased from BD Biosciences, San Jose, CA/USA. cDNA synthesis kit and SYBR Green master mix were obtained from TaKaRa Biotechnology, Japan, and Ampliqon A/S (Odense, Denmark) respectively. Human Phospho-Histone H2AX (γ-H2AX) in cell ELISA kit was purchased from R&D Systems Europe Ltd., Abingdon, UK (Catalog No: DYC2288-2). BRCA1 detection ELISA kit was procured from Biocompare (South San Francisco, CA/USA) (Catalog No: MBS457981). Mre11A Elisa Kit was obtained from Mybiosource (San Diego, CA/USA) (Catalog No: MBS9336466) and Rad51 detection assay kit was purchased from Antibodies-online.com GmbH (Aachen, Germany) (Catalog No: ABIN481732). Specific primers for *BRCA1*, *Rad51,* and *Mre11* were synthesized by Pishgaman Inc. (Tehran, Iran). 

DU145 and PC-3 human Pca cell lines were obtained from the Research Institute of Biotechnology, Ferdowsi University of Mashhad, Iran and validated negative against mycoplasma contamination. Cells were cultured in 10 % FBS-contained RPMI1640 media, streptomycin (100 U/ml), penicillin (105 mg/ml) and Amphotericin B (2.5 mg/mL). After growing to confluency in a humidified atmosphere, 37 °C and 5 % CO_2_ cells were exposed to 0.01 % DMSO, VPA and the combination of VPA and AZD2461. 

### Trypan blue dye exclusion assay

Cells were 60000 cells per well (in 24-well plates). After overnight incubation, the culture medium was carefully discarded and replaced fresh medium with increasing concentrations of VPA (0.5-16 mM) and AZD2461 (5-160 μM). Following 48 hours of incubation, cells were trypsinized, and the pellets were washed with phosphate-buffered saline (PBS). Then, 20 µL of trypan blue solution (0.4 % in PBS) was added to 20 µL of cell suspensions and using a dual-chamber hemocytometer and a light microscope. By dividing the number of unstained (living) cells by the total number of cells, the viability percentage was calculated. 

### MTT cytotoxicity assays and drug combinations analysis

For determining the biochemical half maximal inhibitory concentration (IC_50_) of both inhibitors using MTT cytotoxic assay, both cells were exposed to single agents at increasing concentrations (from 0.5 mM -16 mM for VPA and 5 μM-160 μM for AZD2461). After 48 hours, 20 µL of Tetrazolium dye (5 mg/ml dissolved in serum-free culture media) was added to each microwell and incubated for 3 hours at previously described standard condition. The culture medium was cautiously removed and with 200 μL of DMSO was added to each well, and the absorbance at 570 nm wavelength was measured using a multi-well plate reader (Stat-Fax-2100; Awareness Technologies, Westport, CT/USA). The results were expressed as the absorbance of treated cells divided by the absorbance of adjusted untreated cells × 100.

For experiment with VPA and AZD2461 according to the strongest concentration-dependent effects of the single agents, cells were simultaneously exposed to both drugs (as a single agent) at the following concentration ranges: VPA: 0.25 mM - 32 mM; AZD2461: 3.125 μM - 400 μM (with combination ratio of 1:80 for PC-3 cells) and VPA: 0.125 mM - 16 mM; AZD2461: 3.125 μM - 400 μM (with combination ratio of 1:40 For DU145 cells). Increasing concentrations, each diluted 1:2, within the given ranges were used. CompuSyn software (Version 1.0, Combo-Syn Inc., US) was used to determine the interaction between fixed ratios of VPA and AZD2461 based on the Chou-Talalay method for drug combinations, with CI>1.1, CI= 0.9-1.1 and CI<0.9 indicating antagonistic, additive and synergistic manners, respectively (Chou and Talalay, 1984[7]). Then, the combination index (CI) values were estimated for each level of fraction affected (Fa) according to the following equation: CI = (D)_1_(Dx)_1_ + (D)_2_/(Dx)_2_ + (D)_1_(D)_2_/(Dx)_1_(Dx)_2_, where (D)_1_ and (D)_2_ represent the concentration of AZD2461 and VPA when used in combination and (Dx)_1_ and (Dx)_2_ are the concentrations of mentioned agents when used alone. Also, median-effect principles for drug combination were plotted. 

### Flow-cytometric determination of apoptosis

250000/well of PC-3 and DU145 cells were seeded (in 6-well plates) and exposed to AZD2461 and VPA (equal to IC_50_ values), and their combination for 48 hours. After treatment, cells were resuspended in cold PBS. Cells were then centrifuged at 1500 rpm for 10 minutes and washed again and resuspended in 1X binding buffer followed by adding 5 μL of FITC Annexin V to the cell suspension. After 15 minutes of dark incubation, 5 μL of propidium iodide (PI) was added and incubated for 10 minutes away from light. Then, cell pellets were slowly pipetted while 400 μL of 1X buffered isotonic solution was added to each sample before being analyzed by a Partec PAS flow cytometer (Partec, Münster, Germany). The levels of apoptosis were expressed as percentages of Annexin V^+^/PI^+^ (late apoptosis), Annexin V^+^/PI^-^ (early apoptosis) and Annexin V^-^/PI^+^ (necrosis) as a minimum of 5000 cells was collected for analysis by Flowmax software.

### RNA isolation and real-time PCR analysis

Following 48 hours of treatment of both cell lines with concentrations equal to IC_50 _values of both agents and their combination, extraction of total RNA was performed RNX-Plus Solution (SinaClon Co., Tehran, Iran). For assessment of RNA purity and quantity, 260/280 nm ratios were measured. The synthesis of cDNA was conducted using the cDNA synthesis kit (TaKaRa Biotechnology, Japan) as described by the manufacturer. Briefly, 4 μg of RNA, 2.5μL RNase free dH_2_O and an equal volume of oligo dT Primer and Random 6mers (0.5 μL of each) were mixed in a microtube. Following 5 minutes of incubation at 65 °C and immediate cooling on ice, 0.5 μL of PrimeScript^TM^ RT Enzyme Mix I and 2 μL of 5X PrimeScript^TM^ Buffer were also added to a total volume of 10 μL. PCR amplification was done for 15 minutes at 37 °C, followed by 5 seconds at 85 °C and 10 minutes of holding at 4 °C. 

The primers (shown in Table 1(Tab. 1)), were designed by OLIGO 4.0 software (National Biosciences, Plymouth, MN), spanning exon/intron junctions to avoid amplification of DNA sequences while assuring all the isoforms of four target genes (transcription variants 1 and 2 of *PTEN*, 1-4 of *BRCA1*, 1-3 of *Rad51* and variants 1, 2, 3, X1, X3, X4, X6 of *Mre11*) would be amplified. Using SYBR Green and an ABI Prism 7700 Sequence Detection System (Applied Biosystems, Foster City, CA/ USA), the real-time PCR analysis was performed. PCR conditions were set as follows: 10 minutes/95 °C for initial denaturation followed by 40 cycles each consisted of denaturation for 30 seconds at 95 °C; annealing for 30 seconds at specific annealing temperatures (Table 1(Tab. 1)) and extension for 45 s at 72 °C. Fold changes in mRNA levels of assayed genes were calculated using the comparative 2**^-ΔΔCt^** method. 

### Quantification of H2AX phosphorylation (γ-H2AX)

Following the manufacturer's protocol, γ-H2AX levels were measured by use of a commercially available human phospho-histone H2AX (S139) in cell ELISA kit, an advantageous high-throughput microscopy-based method (Ji et al., 2017[16]) within 3, 12 and 24 hours after treatment with IC_50 _values of single drugs and their combination for both cells. Briefly, the capture antibody was diluted in PBS and instantly coated to a microplate and incubated for 24 hours at room temperature. After repeated washings, 300 μL of block buffer was added and kept at room temperature for 90 minutes. The plate was then aspirated, and the cell lysate/standard was added. The plate was then tightly sealed and kept for 2 hours at 37 °C. Following aspiration and 3 times washing, 100 μL of the diluted detection antibody was placed to each microwell before incubating for 2 hours at 25 °C. Next, 100 μL of the diluted streptavidin-HRP was added, and after 20 minutes of room temperature dark incubation at 37 °C, the aspiration/ washing step was repeated. In the end, 50 μL of stop solution was added, and optical density values for each sample was evaluated using a microplate reader (Stat-Fax-2100; Awareness Technologies, Westport, CT/ USA) directly at 450 nm. In cell levels of γ-H2AX were expressed as pg/mL in a time-dependent manner.

### Detection of protein levels of BRCA1, Mre11A, and Rad51

After seeding PC-3 and DU145 cells (250000/well) followed by overnight incubation, both cells were exposed to IC values of both agents alone and in combination before 48 hours on incubation. Then, the protein levels of three HR-mediated DSB repair factors including BRCA1, Mre11A and, Rad51 are measured using standard enzyme-linked immunosorbent assay (ELISA) kits, according to manufacturer's protocols, and the results were expressed as pg/mL. A standard curve with at least seven points was plotted for each assay. 

### Statistics

Using the sample T-test, non-parametric ANOVA, and the Dunnett post hoc test, the results were analyzed via SPSS16 software (release 16, SPSS Inc., Chicago, IL/USA). p-Values less than* <0.05* were regarded as statistically significant while all assays were repeated at least three times. 

## Results

### Effects of VPA and its combination with AZD2461 on Pca cell lines

**Figure 1:**
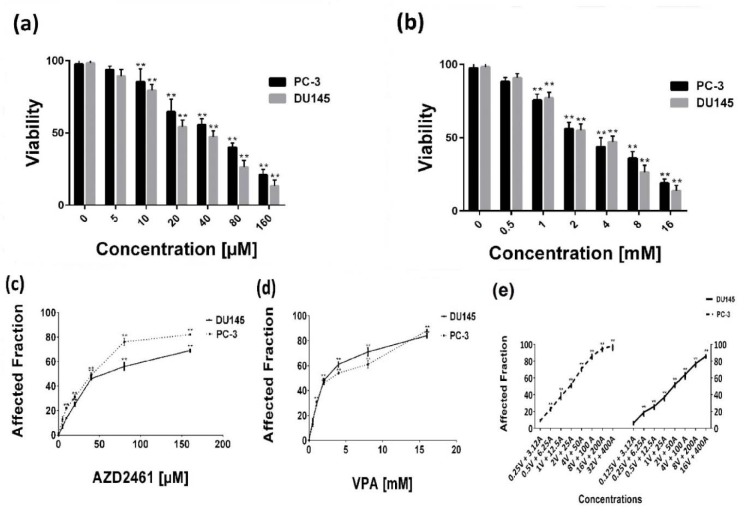
Concentration-response effects of AZD2461 and VPA on PC-3 and DU156 cells using (a, b) trypan blue exclusion assay and (c, d) MTT assay following 48-hours treatment with both agents separately. (e) AZD2461 and VPA combination after 48 hours post treatment against PC-3 and DU145 cell lines depicted by GraphPad Software.(**P<0.05 compared with control)

Within the described ranges, both drugs evidently decreased the viability of both cells in a concentration-dependent manner (Figure 1a, b, c, and d(Fig. 1)), verified by both MTT and trypan blue dye exclusion methods (*P<0.05*). The IC_50_ values of VPA and AZD2561 calculated by CompuSyn software were 3.07 mM and 38.48 µM for PC-3 cells and 2.47 mM and 61.51 µM for DU145 cells, respectively. PC-3 were found to be more responsive to PARP inhibition by AZD2461 while DU145 cells were more sensitive to VPA. 

**Figure 2:**
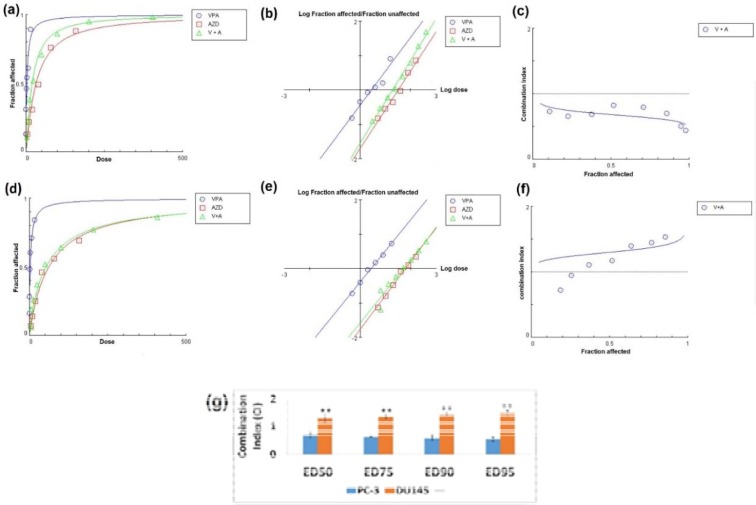
Analysis of synergy between VPA and AZD2461. (a), (b) and (c) represent dose effect-plot, median-effect plot and combination index plot respectively for PC-3 cells while (d), (e) and (f) are indicative of these three plots for DU145 cells. (g) Different effective doses (EDs) of VPA and AZD2461 combination in both cell lines (**p < 0.05 compared PC-3 cells)

Later, MTT results indicated that although the combination of two agents mediated synergistic effects PC-3 cells (CI<0.9) (Figure 2a, b, and c(Fig. 2))*, *this regimen showed mild antagonistic effects on DU145 cell line (CI>1.1) (Figure 2d, e, and f(Fig. 2)). Different effective doses (EDs) of VPA and AZD2461 combination in both cell lines are shown in Figure 2g(Fig. 2).

### Combination of VPA and AZD2461 induces marked apoptosis of PC-3 cells

**Figure 3:**
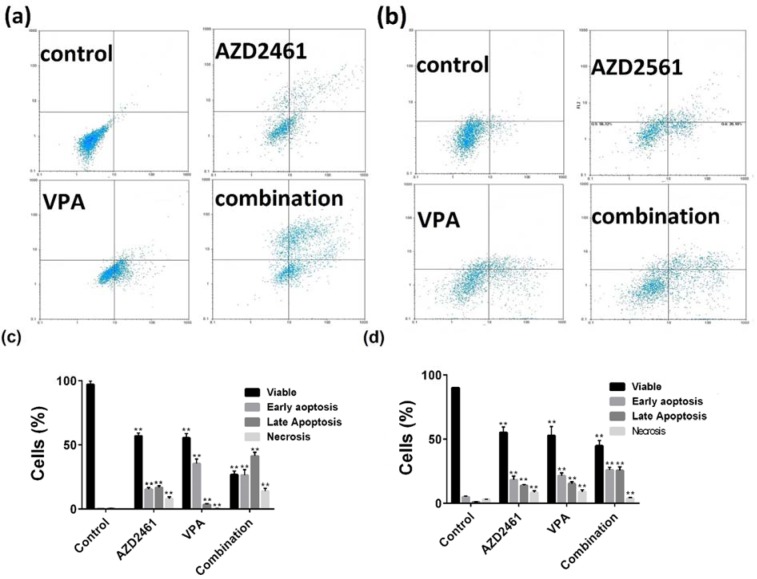
Flow cytometry of apoptosis by Annexin V and propidium iodide (PI) double staining. (a, c) DU145 and (b, d) PC-3 cells were treated with VPA and AZD2461 for 48 hours. Following the treatment with IC_50_ values of both inhibitors on both cell lines, the cells were harvested and apoptosis was assessed (**p < 0.05 compared with control)

Annexin V/PI double staining results indicated that total apoptotic portion of PC-3 cells treated with both inhibitors and their combination were 41.9 %, 34.3 %, and 62.7 % respectively (Figure 3a, c(Fig. 3)), while these portions were 34 %, 34.1 %, and 41.8 % for DU145 cells (Figure 3b, d(Fig. 3)). As shown in Figure 3(Fig. 3), although the combination of VPA and AZD246 caused higher apoptosis rates than each component alone, significantly higher levels of cell death were observed in PC-3 cells treated with the combination of these agents compared to DU145 cells (*P<0.05*). 

### Effect of VPA, alone or in combination with AZD2461, on BRCA1, Rad51 and Mre11 mRNA levels

**Figure 4:**
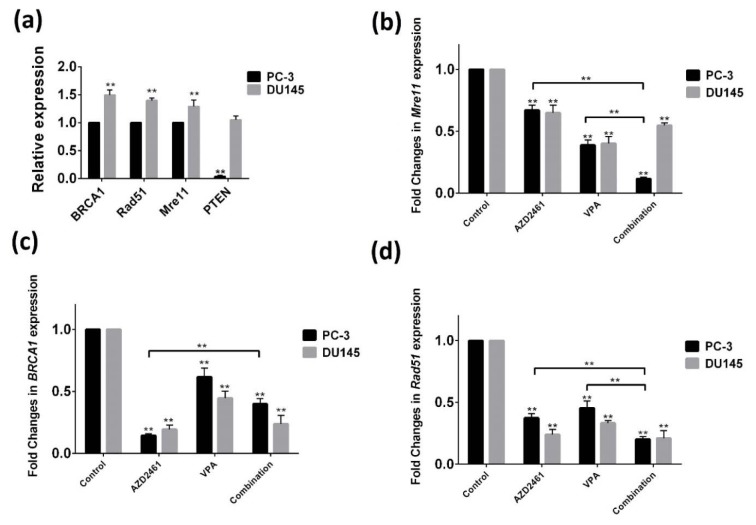
Real-time PCR method was employed to determine the mRNA level of HR-associated genes. Basal mRNA levels of *BRCA1*, *Mre11*, *Rad51*, and *PTEN* in PC-3 cells compared to DU145 cells indicates that DU145 cells have a higher basal expression of these genes (a). The measured mRNA levels of *Mre11 *(b), *BRCA1 *(c), and *Rad51 *(d) genes in both cells treated for 48 hours with AZD2461, VPA and their combination has revealed that these regimen was only effective in reducing Mre11 and Rad51 expression in PC-3 cells. Each assay was normalized to housekeeping gene *GAPDH* (**p < 0.05).

While PC-3 cells displayed significantly lower *PTEN* expression levels compared to DU145 cells, both PC-3 and DU145 cells displayed a baseline expression of three genes with significantly higher expression levels in DU145 cells (*p<0.05*). As shown in Figure 4a(Fig. 4), AZD2461 significantly down-regulated the mRNA levels of *BRCA1*, *Rad51* and *Mre11* (at 87, 68 and 33 % of the control in PC-3 cells and 83, 79 and 35 % of control in DU145 cells respectively) following 48 hours of treatment. Additionally, VPA was found to be able to decrease the expression levels of *BRCA1* (33 % of the control in PC-3 cells and 59 % in DU145 cells), *Rad51* (55 % of the control in PC-3 cells and 69 % in DU145 cells) and *Mre11* (61 % of the control in PC-3 cells and 60 % in DU145 cells). Real-time PCR results also revealed that although when PC-3 cells were exposed to co-treatment of VPA and AZD2461, the mRNA levels of *Mre11* and *Rad51* were significantly decreased compared to both drugs individually (*P<0.05*) (Figure 4b-d(Fig. 4)), but reduction in mRNA levels of *BRCA1* was less marked (Figure 4c(Fig. 4)). Also, the decreases in *BRCA1*, *Rad51* and *Mre11* mRNA levels seen with the combination of VPA and AZD2461 in DU145 cells were not significant to those anticipated from the activity of the single agents.

### VPA combined with AZD2461 increases γ-H2AX levels more than AZD2461 alone in PC-3 Cells

**Figure 5:**
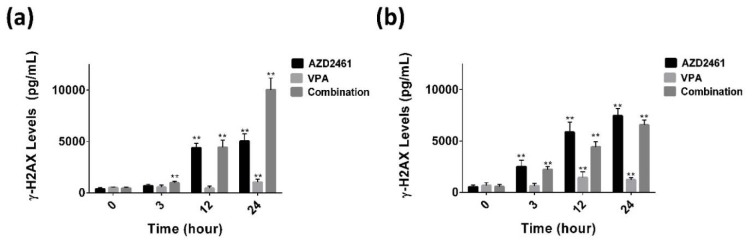
Phosphorylation of H2AX was detected using enzyme-linked immunosorbent assay following 3 to 24 hours of exposing PC-3 (a) and DU145 cells (b) to VPA, AZD2461 and their combination. Following 12 and 24 hours, a significant increase was observed in γ-H2AX levels when PC-3 cells were treated with AZD2461+VPA (**P<0.05).

As shown in Figure 5a(Fig. 5), following 3, 12 and 24 hours treatment of PC-3 cells with the combination of AZD2461 and VPA, the levels of γ-H2AX were significantly increased about 1.7, 3.6 and 5.7 fold respectively compared to adjusted untreated cells, but these values were 2, 2.4 and 2.9 for DU145 cells (Figure 5b(Fig. 5)*)*. Although after 3 hours treatment, an increase in γ-H2AX levels was observed in both cells co-treated with both agents, this combination regimen did not significantly enhance H2AX phosphorylation of DU145 cells at 12 and 24 hours treatment periods compared to each agent alone (*P<0.05*). This data suggests that significant phosphorylation of H2AX was observed in all experimental groups of PC-3 cells at all treatment period (*P<0.05*).

### The outcome of combination therapy on protein levels of HR-mediated DSB repair factors

**Figure 6:**
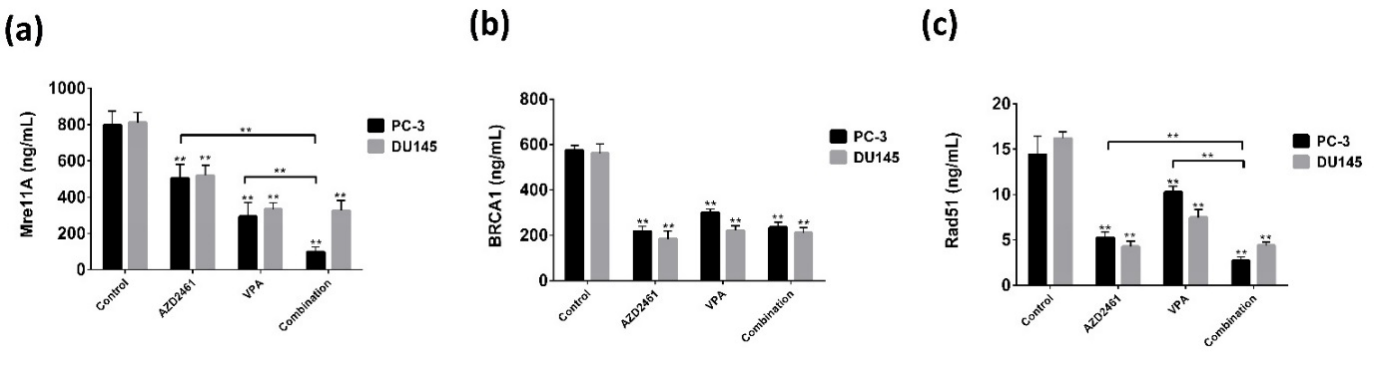
Enzyme-linked immunosorbent assay was used to determine the protein levels of HR-associated factors. The measurement of the protein levels of Mre11 (a), BRCA1 (b), and Rad51 (c) in both cells treated for 48 hours showed that the levels of BRCA1 was not affected by AZD2461+VPA, while this combination decreased Mre11 and Rad51 levels only in PC-3 cells (**P<0.05).

As participated and consistent with our findings in regards of the mRNA levels of BRCA1, Mre11 and Rad51, we only observed that VPA+AZD2461 was only able to significantly decrease the protein level of Mre11 and Rad51 (*P<0.05*) (Figure 6a, c(Fig. 6)) compared with cells treated with AZD2461 or VPA. No changes in protein levels of BRCA1 was observed when applying this regimen on PC-3 and DU145 cells (*P>0.05*) (Figure 6b(Fig. 6)).

## Discussion

Usually, in prostate tissue, cell growth and proliferation are regulated by apoptotic cell-death mechanism, but in Pca cells, an imbalance in this process occurs, causing the tumor cells to progress (Denmeade et al., 1996[9]). Therefore, the objective of targeted therapy for this kind of malignancy is to compensate for this imbalance. Regrettably, effective restoring of apoptotic balance in high-grade Pca cells has not been yet accomplished as metastatic Pca still considers as a fatal disease with no curative therapy. Hence, understanding of the cell-death related pathways and learning about how to control them are required for achieving such efficacious therapies.

PARP1 role in HR pathway has been recently gained much attention due to attempts towards the development of potent and selective PARP1 inhibitors to suppress DNA repair pathways and induction of cell death in cancer cells with perturbed DNA damage response. Although the exact mechanism of *PTEN* involvement in HR pathway is not entirely understood, it is believed to be essential for recruiting Rad51 to DSB sites and while interacting with BRCA1 (McEllin et al., 2010[26]). As we determined of IC_50 _values of both drugs, our data indicated that even though PC-3 cells are more sensitive to PARP inhibition by AZD2461, but VPA has higher cytotoxic effects on DU145 cells. Both PC-3 and DU145 cells displayed a basal expression of HR gene products (Rauh‐Adelmann et al., 2000[34]), but they varied in their *PTEN* expression status as DU145 displayed higher mRNA levels of *PTEN*. The precise anti-neoplastic mechanism of action for HDAC inhibitors, such as VPA, has not been completely understood, but their role in down-regulating proteins of NHEJ and HR pathways in cancer cells are well explained (Munshi et al., 2006[29]). Although the exact effects of *PTEN* insufficiency on DNA repair signaling pathways is ill-defined, McEllin et al. declared that *PTEN*-null astrocytes express significantly lower levels of *RAD51* (McEllin et al., 2010[26]), indicating *PTEN* expression is essential in order to maintain the basal expression of RAD51 (Shen et al., 2007[36]). *RAD51* gene family has been reported to be widely expressed in high-grade Pca (Gleason score >7) and correlates with reduced patient survival (Chao and Goodman, 2014[6]). These findings suggest *RAD51* suppression as a potential target in the treatment of cancer cells overexpressing this gene. Also, a decrease in Mre11 mRNA levels was found to be associated with *PTEN *expression level status of HCT116 cells (Xu et al., 2010[40]). 

Due to their broad range of targets, it is possible that VPA may have affected the function of other proteins involved in DNA damage response. The cytotoxic effects of HDAC and PARP inhibitors as monotherapeutic agents on Pca cells is reported to be minimal (Chao and Goodman, 2014[6]), thus highlighted the need for developing combination strategies. Drug interaction between VPA and AZD2461 in Pca cells with different *PTEN* expression level status has not yet been elucidated. Our results suggest a possible synergistic relationship between VPA and AZD2461 but only in PC-3 cells (CI<0.9) while we observed mild antagonism (CI>1.1) in DU145 cells which were in contrast with Chao's and Goodman's experiment reporting that synergistic potential of combination therapy of HDACi and PARPi is observed mainly in DU145 cell lines expressing high levels of *PTEN* (Chao and Goodman, 2014[6]). Konstantinopoulos et al. (2014[20]) were hypothesized that relative olaparib resistance could be overcome by a combination of olaparib with SAHA. This combination regimen could down-regulate the expression levels of *RAD51* and *BRCA* as HR-related genes besides displaying a synergistic enhancement of cytotoxicity in ovarian cancer cell lines with functional and non-functional DNA repair systems. Our data indicate that co-treatment of AZD2461 and VPA led to a robust down-regulation of *RAD51 *and* Mre11* compared to the modest effect by the inhibitors individually as down-regulation of RAD51 protein levels in Pca cells due to PARP inhibition was reported earlier in a study conducted by Hegan et al. (2010[12]). In agreement with the findings of Minami et al. experiment, we observed lower basal expression of *Mre11* in PC-3 cells compared to DU145 cells (Minami et al., 2013[28]). The mRNA levels of *BRCA1* was found to be less affected with combination therapy of both inhibitors, recommending a possible explanation of that *BRCA1* might not be critical in preventing DNA-damage induced apoptosis (Martin et al., 2007[25]). Likewise, Annexin-V staining analysis results indicated substantial apoptosis upon treatment of PC-3 cells with VPA+AZD2461 compared to DU145 cells. Although we observed elevated necrosis rate just in PC-3 cells when co-treated with both inhibitors, this is not entirely in agreement with Chao's observations declaring other cell death pathways (i.e., necrosis) might be the primarily responsible mechanism for the reduction in PC-3 cell viability when combining HDAC and PARP inhibitors. We also observed that VPA can significantly down-regulate *BRCA1*, *Rad51,* and *Mre11* in both PC-3 and DU145 cells which were not different from Kachhap and coauthors' (2010[17]) experiment, but the reduction in expression levels of these three genes in HDACi and PARPi-treated cells are consistent with findings of other similar studies (Chao and Goodman, 2014[6]; Min et al., 2015[27]).

Furthermore, like Shen and his colleagues argued, in case of exposure to either ionizing radiation or DNA-damaging agents, ATM and related protein kinases phosphorylate the carboxyl-terminal end of the H2AX protein and form γ-H2AX (Shen et al., 1998[35]). In such stress conditions, maximum accumulation of γH2AX, as a marker of DNA damage occurs (Ji et al., 2017[16]) as BRCA1 carboxyl-terminal (BRCT) domain-containing proteins are recruited to γ-H2AX in order to form a protein scaffold for assembling other signaling complexes (Yan and Jetten, 2008[41]). Significant and persistent increase in nuclear γH2AX levels is reported in many experiments regarding the combination of PARP inhibitors with other DNA damaging agents, although these agents were not capable of altering γH2AX levels alone (Chao and Goodman, 2014[6]; Min et al., 2015[27]). Consistent with these findings, only in PC-3 cell lines in which a synergistic effect was observed, we found increased levels of in cell γ-H2AX, following 3, 12, and 24-hours treatment with VPA and AZD2461. We then measured protein levels of HR-mediated DSB repair factors. Unsurprisingly, we observed that combination of AZD2461+VPA is unable of significantly change the protein levels of BRCA1 in both cell lines, providing data supporting the hypothesis that Pca cells might be less dependent to BRCA1-mediated DNA repair, while the other two measured factors were significantly altered by use of this regimen. This was in contrast with findings of Fraser et al., declaring that it is unlikely that *PTEN* expression status will be an appropriate biomarker for HR status or PARPi response in Pca clinical trials (Fraser et al., 2012[11]), suggesting that the correlation between *PTEN* status and cell survival caused by DNA damaging agents is more complicated than it seems. 

In the present study, we assessed the combination effects between a potent and well tolerated PARPi, AZD2461, and a HDACi, VPA in two Pca cells with different genetic backgrounds in terms of *PTEN* expression. Although our results exhibit a synergistic relationship of VPA and AZD2461 in PC-3 cells, harboring a heterozygous deletion of *PTEN*, these findings require further investigations. Evaluation of combined effects of AZD2461 and other DNA damaging agents (i.e., alkylating agents) might be pursued in clinical trials.

## Conclusion

The outcome of Pca treatment mainly depends on a molecular understanding of the patients' genetic backgrounds as recently *in vitro *and* in vivo *studies suggested that specific tumor cells would respond more effectively to selective inhibitors if carrying a mutation affecting their DNA repair efficiency. The current study was designed to investigate the possible link between *PTEN* status of two Pca cell lines and sensitivity to the combination of PARPi+HDACi. By performing MTT, flow cytometric analysis, real-time PCR, and in cell ELISA assays, we concluded that using this regimen might be more applicable to PC-3 cells lacking *PTEN* expression. Our data provide proof-of-principle of the effectiveness of a strategy for combining VPA and AZD2461 to treat Pca patients with *PTEN*-backgrounds. Knowledge of other HR-related genes profile may discriminate patients who would respond more effectively to such combination strategies.

## Notes

Saman Sargazi and Ramin Saravani (Cellular and Molecular Research Center and Department of Clinical Biochemistry, School of Medicine, Zahedan University of Medical Sciences, Zahedan, Iran; E-mail: saravaniramin@yahoo.com, Tel: +989155432609, Fax: +985433295731) contributed equally as corresponding authors.

## Acknowledgements

This work is financially supported by Shahid Sadoughi University of Medical Sciences, Yazd, Iran (Grant No: 5734). 

## Conflict of interest

None declared.

## Figures and Tables

**Table 1:**
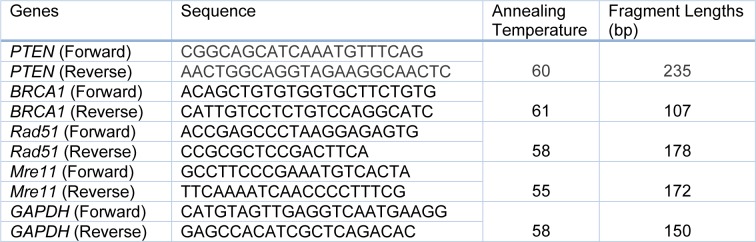
Primer sequences of *PTEN*, *BRCA1*, *Rad51*, *Mre11* and *GAPDH*
